# Alpha 1-Antitrypsin Does Not Inhibit Human Monocyte Caspase-1

**DOI:** 10.1371/journal.pone.0117330

**Published:** 2015-02-06

**Authors:** Mohd. Akhlakur Rahman, Srabani Mitra, Anasuya Sarkar, Mark D. Wewers

**Affiliations:** Dorothy M. Davis Heart and Lung Research Institute, Department of Internal Medicine, Division of Pulmonary, Allergy, Critical Care and Sleep Medicine, Wexner Medical Center, Ohio State University, Columbus, OH, United States of America; University of Tübingen, GERMANY

## Abstract

**Background:**

Alpha 1-antitrypsin (A1AT) is a 52 kDa serine protease inhibitor produced largely by hepatocytes but also by mononuclear phagocytes. A1AT chiefly inhibits neutrophil elastase and proteinase-3 but has also been reported to have immune modulatory functions including the ability to inhibit caspases. Its clinical availability for infusion suggests that A1AT therapy might modulate caspase related inflammation. Here we tested the ability of A1AT to modulate caspase-1 function in human mononuclear phagocytes.

**Methods:**

Purified plasma derived A1AT was added to active caspase-1 in a cell-free system (THP-1 lysates) as well as added exogenously to cell-culture models and human whole blood models of caspase-1 activation. Functional caspase-1 activity was quantified by the cleavage of the caspase-1 specific fluorogenic tetrapeptide substrate (WEHD-afc) and the release of processed IL-18 and IL-1β.

**Results:**

THP-1 cell lysates generated spontaneous activation of caspase-1 both by WEHD-afc cleavage and the generation of p20 caspase-1. A1AT added to this cell free system was unable to inhibit caspase-1 activity. Release of processed IL-18 by THP-1 cells was also unaffected by the addition of exogenous A1AT prior to stimulation with LPS/ATP, a standard caspase-1 activating signal. Importantly, the A1AT exhibited potent neutrophil elastase inhibitory capacity. Furthermore, A1AT complexed to NE (and hence conformationally modified) also did not affect THP-1 cell caspase-1 activation. Finally, exogenous A1AT did not inhibit the ability of human whole blood samples to process and release IL-1β.

**Conclusions:**

A1AT does not inhibit human monocyte caspase-1.

## Introduction

Alpha 1-antitrypsin (A1AT) is an acute phase glycoprotein primarily synthesized and secreted by hepatocytes [1]. Its major function is the inhibition of neutrophil elastase (NE) [2,3]. In A1AT deficiency, low level of plasma A1AT are believed to allow neutrophil elastase unfettered access to lung connective tissue inducing pulmonary emphysema. But A1AT is also actively transcribed and secreted in relatively smaller amounts by cells including neutrophils, mononuclear phagocytes, enterocytes [4,5], and human respiratory epithelial cells [6]. Although the primary role of A1AT is to inactivate neutrophil elastase [2], it may have other pathobiologically relevant functions. A1AT may not only provide protection against proteolytic injury, but it may also exert anti-apoptotic functions. It has been reported that A1AT can rescue serum withdrawal-induced apoptosis [7] and inhibition of structural alveolar cell apoptosis in emphysema [8]. In overexpression studies in THP-1 cells cytosolic A1AT blocks IL-1β release implying an effect on caspase-1 function [9]. Furthermore, A1AT has been proposed to have caspase inhibitory activity [7,8]. More specifically recent studies have suggested that A1AT directly inhibits caspase-1 activation [9,10].

Intracellular proteases such as caspases are responsible for executing mammalian cell apoptosis. In this context, caspase-1 modulates inflammatory responses to pathogen challenge, ischemia and tissue injury and it can promote a form of apoptosis called pyroptosis [11,12]. Moreover, caspase-1 has a unique role because it activates proinflammatory cytokines interleukin (IL)-1β and IL-18 during inflammasome activation. A classical means to activate the monocyte/macrophage caspase-1 inflammasome is via priming by lipopolysaccharide (LPS) followed by induction of K^+^ efflux with exogenous ATP which results in the release of mature IL-1β and IL-18 [13–15].

We previously noted that A1AT mimics proIL-1β in its amino acid sequence and by extrapolation we hypothesized that A1AT may serve as a model of proIL-1β’s structure [16]. Furthermore, monocytes activated by LPS release increased amounts of A1AT that appears to be complexed to an unidentified protease [17] and it is now well accepted that activated monocytes release caspase-1 [18,19]. Therefore, it is reasonable to hypothesize that A1AT may directly interact with and inhibit caspase-1. To address this possibility, we tested whether clinical grade A1AT can affect endogenous caspase-1 activation in various experimental conditions. Our approach used various models of caspase-1 function in order to test A1AT’s ability to prevent activation as well as to inhibit preformed caspase-1. We used the highly concentrated cell-free lysate model of caspase-1 activation [20,21], and the classic models of LPS treatment followed by ATP in an *in vitro* cell culture system[15,22] as well as whole blood models of LPS induced caspase-1 activity[23].

## Materials and Methods

### Cells and Reagents

THP-1 cells were purchased from American Type Culture Collection (lot 385653) and confirmed to be free of mycoplasma prior to use [24]. Human PBMCs were isolated by Histopaque density gradients from fresh source leukocytes from the American Red Cross. Monocytes were isolated from PBMCs by CD14^+^ selection (Miltenyi Biotec). In brief, blood was layered on lymphocyte separation medium (Cellgro) and spun at 600 X g for 20 min at room temperature with brakes off. The mononuclear layer was collected and washed three times with RPMI 1670. Monocytes were purified from PBMCs using positive selection with anti-CD14-coated magnetic beads, following the manufacturer’s recommendations (Miltenyi Biotec). This method of purification yields >98% pure monocytes based on flow cytometry analysis. THP-1 cells and monocytes were cultured in RPMI 1640 (Media Tech) supplemented with 10% heat-inactivated FBS (Atlanta Biologicals) and 1% penicillin-streptomycin (Invitrogen Life Technologies). FBS lots were prescreened to confirm that they did not induce IL-18 release by ATP in the absence of LPS. Ultra-pure LPS from *Escherichia coli* strain 0111:B4; Alexis Biochemicals. ATP, bovine serum albumin BSA, MeOSuc-Ala-Ala-Pro-Val chloromethyl ketone (MeOSuccinyl-AAPV-CMK) and human neutrophil elastase substrate MeOSuccinyl-AAPV-PNA were from Sigma-Aldrich. Alpha-1-proteinase inhibitor (A1AT) was obtained from the clinical pharmacy (Prolastin), Human neutrophil elastase was from EMD Millipore, IL-18 antibody from MBL Medical and Biological Laboratories Co., Ltd., caspase-1 antibody (in house), caspase-1 substrate (WEHDafc) from Calbiochem and caspase-1 inhibitors (YVADcmk) were from EMD Chemicals, Inc. USA.

### Cell-Free Experiments

THP1 cells were lysed at a concentrations of 3x10^7^ cells/100 μl in a hypotonic buffer (Buffer W: 20 mM HEPES, 10 mM KCl, 1.5 mM MgCl_2_, 1 mM EDTA, 1 mM EGTA, pH = 7.4). Briefly, after washing the cells in 1 ml cold PBS, the cell pellet was resuspended in 100 μl of cold Buffer W supplemented with complete protease inhibitor cocktail, 1mM PMSF, and 100 μM AAPV-cmk, and allowed to swell on ice for 10 minutes under these hypotonic conditions. Lysis was accomplished by 10 slow strokes using a 28½g needle on a tuberculin syringe while on ice. Cells were spun at 16,000g for 15 minutes at 4°C in a bench top centrifuge to remove nuclei and large subcellular structures. Supernatants were placed into pre-chilled Eppendorf tubes for caspase-1 cleavage and activity assays.

### Caspase-1 activity assay

Cleavage of the caspase-1 fluorogenic tetrapeptide substrate (WEHD-afc) was used to assess functional caspase-1 activity. Briefly, 50 μl of sample was added to 50 μl of assay buffer (Buffer W + 100 μM WEHD-afc + 10 mM DTT) in a 96-well black Costar plate (Fischer Scientific) and activity was measured using a spectrofluorometer CytoFluor Series 4000 Fluorescence Multi-Well Plate Reader (excitation 360/40 nm, emission 460/40 nm). Readings were taken every 30 seconds or every minute for 60–120 min and slopes were calculated over the linear portion of the curves and expressed as arbitrary fluorescent units.

### Neutrophil elastase assay

To confirm the function of A1AT used in this study on elastase activity, the anti-neutrophil elastase capacity was measured by a modification of the technique of Meyer et al [25]. Increasing amounts of A1AT were incubated with 3 nM of neutrophil elastase in a volume of 1 mL of 0.1 M Hepes, pH 7.5, 0.5 M NaCl, 0.1% Brij. Samples were incubated for 1 h, i.e., more than five times the expected *t*_*1/2*_ for the interaction (overnight incubations did not change the curves). Residual elastase activity was assayed by the addition of 0.1 mM MeOSuccinyl-AAPV-CMK and recording the change of absorbance at 410 nm on a Beckman DU-50 spectrophotometer [26].

### Whole blood collection from healthy volunteers

Blood collected from the three healthy subjects not taking any medications by using antecubital venipuncture, aspirated into glass vacuum tubes containing sodium heparin (Becton Dickinson, NJ, USA). Written informed consent was received from each person following our protocol approved by the Ohio State University Wexner Medical Center Institution Review Board

### Human whole blood cultures with clinical grade A1AT

Whole blood from the three healthy subjects was used without or with dilution at 1:32 in RPMI. Blood cultures were performed in the presence of A1AT (2 mg/mL) after dilution at 1:32 in RPMI, with or without LPS (1.0 μg/mL). All final culture was in 1.0 ml volume in 12 × 75 mm snap-cap polypropylene tubes. After 18 h of incubation, plasma components of the blood cultures were separated and frozen at-80°C until assayed.

### IL-1β and IL-18 ELISA

Released IL-1β was quantified with a sandwich ELISA format, using our rabbit polyclonal as previously reported [27] but substituting monoclonal (MAB601) from R&D Systems for the captured antibody. Released IL-18 was quantified by sandwich ELISA using MBL antibodies. Anti-human IL-18 (MBL International, mouse IgG2A monoclonal) was coated on 96-well clear Costar plate (Fischer Scientific) at 1:1000 overnight at 4°C. Plates were blocked with 5% bovine serum albumin, followed by incubation of samples and recombinant IL-18 standard (MBL International). Biotin-labeled anti-human IL-18, 1:1000 (MBL International, rat IgG2A monoclonal) was then added. Each step was incubated for a minimum of 1h with 4 washes using PBS + 0.5% Tween 20. Streptavidin-HRP (eBiosciences) was added for 1h and after washing, the plate was developed using TMB Peroxidase Substrate and Peroxidase Substrate Solution B (KPL, SeraCare Life Sciences). Plates were read on a Perkin Elmer 2030 Victor X3 Multilabel Reader, measuring absorbance at 450 nm after subtracting background 630 nm absorbance.

### Western-blot detection of proteins

For the in vitro caspase-1 and IL-18 analysis, 40 μl of supernatant was loaded onto 4–12% Bis-Tris SDS gel (NuPAGE Novex, Life Technologies) after denaturing the proteins in Laemmli buffer. Gels were run in MOPS SDS buffer (NuPAGE MOPS SDS running buffer (20×), Life Technologies). Magic Mark XP Western Standard (Invitrogen) was used as a molecular weight marker. Proteins were transferred onto PVDF membranes in Tris-glycine buffer (20% methanol, Tris-glycine (10×), Bio-Rad). Membranes were blocked in 10% non-fat dry milk (TBS (20×) + 0.1% Tween 20) or 10% bovine serum albumin (TBS = 0.1% Tween 20) for the biotin-labeling experiments. Anti-caspase-1 and anti-IL-18 antibodies (1:1,000) were used to probe gels, and respective secondary antibodies (1:10,000) were subsequently added. Membranes were rinsed and washed 3 times with TBS + 0.1% Tween-20 between each of the incubation steps. Enhanced chemiluminescence solution (Amersham, GE Life Sciences) was used to detect labeled proteins and blots were developed using HyBlot CL autoradiography film (Denville Scientific Inc.) on a Konica Minolta SRX-101A film processor (Konica Minolta Medical Imaging U.S.A., Inc.).

### Statistics

Student’s t-test was used for comparison between two groups to analyze significant differences. p ≤ 0.05 was considered to be significant.

## Results

### Effect of A1AT on caspase-1 activation in cell-free system

**Fig 1:**
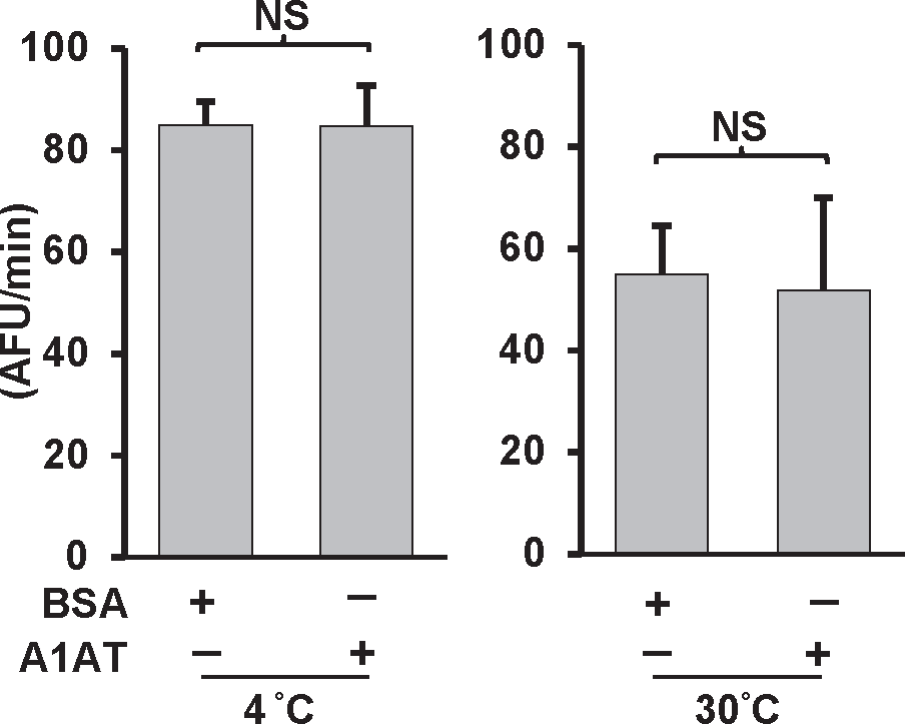
A1AT is ineffective on endogenous caspase-1 activation in cell-free system. Caspase-1 activity was measured in cell-free lysates from THP-1 monocytes. Cell-free lysates in a hypotonic buffer were placed at 4 or 30°C for 1 hour with or without A1AT (2.5 mg/mL). Same concentration of BSA was used as control. Caspase-1 activity was then measured by using caspase-1 fluorogenic tetrapeptide substrate (WEHD-afc) according to materials and methods. Data represent the means ± SD for three independent experiments. NS indicates no significant difference between BSA control and A1AT, as analyzed by Student’s t-test.

Lysing the human monocyte cell (THP-1) at high concentrations in hypotonic buffers induces the spontaneous activation of caspase-1 [28,29]. We therefore used this model system to determine if exogenously added A1AT could inhibit the activity of caspase-1 as measured by cleavage of the caspase-1 substrate, WEHD-afc. We added plasma-derived commercial A1AT (Prolastin) to highly concentrated cell-free extracts containing endogenous caspase-1. Activation of caspase-1 was induced by lysing monocytes at 3 x10^8^ cells/ml and quantified by conversion of a caspase-1 specific fluorescent substrate. We found that endogenous caspase-1 loses almost half of its activity within one hour of incubation at 30°C (as previously shown [30]). However, the presence of A1AT 2.5 mg/ml had no effect on caspase-1 function **(Fig. 1)**.

### Effect of A1AT on caspase-1 activation in cell-culture system

**Fig 2:**
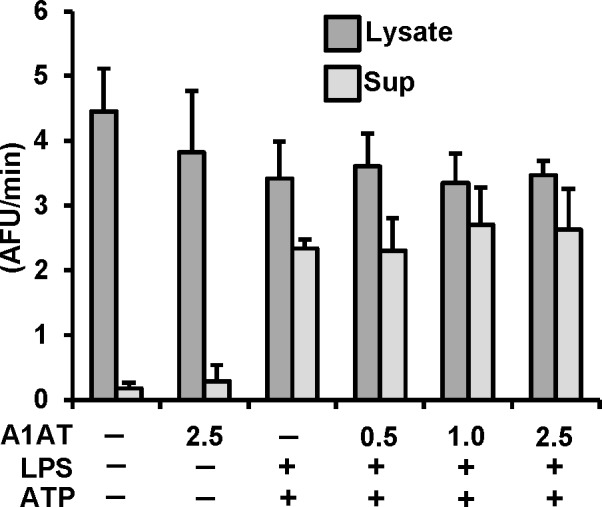
Effect of A1AT on caspase-1 activation in THP-1 cells culture. THP-1 cells pretreated with A1AT (0.5 to 2.5 mg/ml), then LPS 1 μg/ml stimulated for 30 minutes followed by 5 mM ATP challenge for another 30 minutes. Caspase-1 activation was measured both in lysate and supernatant by using caspase-1 fluorogenic tetrapeptide substrate (WEHD-afc) according to materials and methods. Data represent the mean ± SD for three independent experiments.

To rule out the possibility that A1AT has an effect that requires the presence of live cells, we studied the effect of A1AT on caspase-1 activation in THP-1 cells. THP-1 cells were pretreated with A1AT for 1h followed by LPS priming (1 μg/ml) for 30 min and then triggering with ATP (5 mM) for another 30 min to induce caspase-1 activation using our published model [15,31]. As shown in **Fig. 2**, increasing concentrations of A1AT (0.5 to 2.5 mg/mL) had no effect on caspase-1 activity in cell lysates or supernatants compared to controls. In separate experiments this A1AT preparation was confirmed to be effective in inhibiting neutrophil elastase activity on a mole for mole basis (data not shown).

### Release of mature IL-18 in response to LPS-ATP treatment is unaffected by A1AT

**Fig 3:**
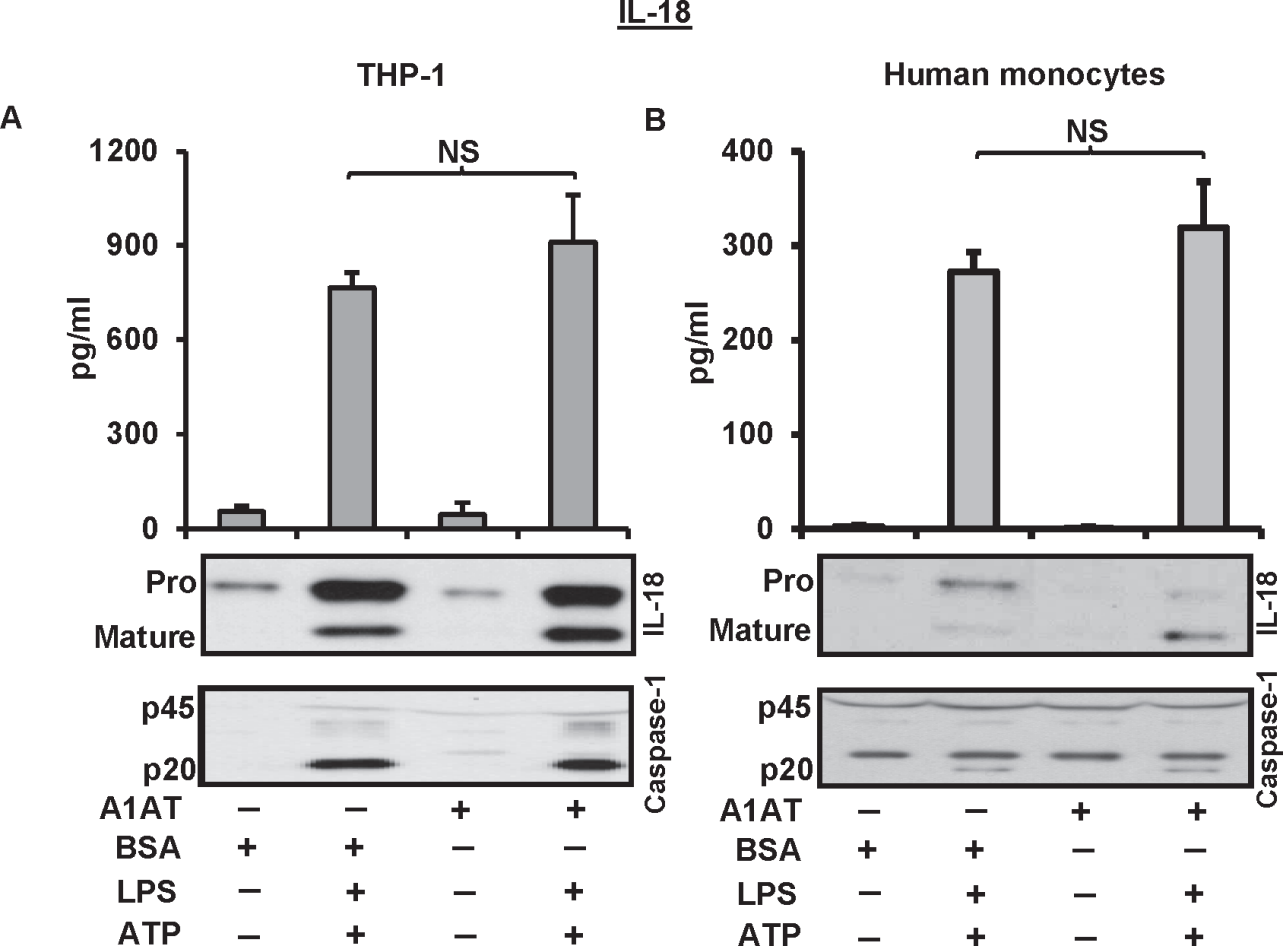
A1AT does not influence IL-18 processing and release through the activation of caspase-1. THP-1 cells and human monocytes were pre-treated with A1AT (2.5 mg/mL) for 1 h followed by LPS 1 μg/ml for 30 minutes, and then triggered with ATP 5 mM for another 30 minutes to determine the release of IL-18 and caspase-1. Same concentration of BSA was used as control. **A.** THP-1 cell and **B.** Human monocytes, culture media were assayed by ELISA for the released IL-18, and by immunoblots for IL-18 and caspase-1. ELISA data represent the means ± SD for three independent experiments in THP-1 cells and human monocytes, and the blots are representative of repeated blots. NS indicates no significant difference between BSA control and A1AT treatment, as analyzed by Student’s t-test.

It is known that LPS/ATP treatment induces release of mature IL-18 through the activation of caspase-1 [15]. Therefore, we studied the ability of A1AT to inhibit IL-18 processing. To do this we treated THP-1 cells and human monocytes with A1AT 1 h before a 30 min pulse of LPS and then ATP for another 30 min to induce the release of mature IL-18. Maturation of IL-18 through the caspase-1 activation was not influenced by A1AT treatment in this experimental model (**Fig. 3A, 3B**). In addition, when we extended these studies to monocyte mRNA expression patterns, we found exogenously added A1AT did not suppress endotoxin-induced *IL1B* and *IL-18* mRNA expression (data not shown).

### Conformationally-modified A1AT does not suppress caspase-1 activation nor IL-18 processing

**Fig 4:**
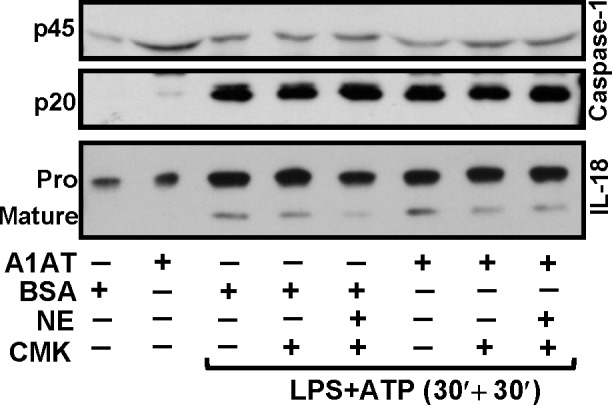
Conformationally modified A1AT does not suppress caspase-1 and IL-18 release. THP-1 cells was pretreated with A1AT-HNE complex at molar ratio of 1:1 for 1 h followed by LPS 1 μg/ml for 30 minutes, and then triggered with ATP 5 mM for another 30 minutes to determine the release of caspase-1 and IL-18. Released caspase-1 (pro and cleaved) and IL-18 (pro and matured) were blotted with caspase-1 and IL-18 specific antibodies, respectively. Released caspase-1 and IL-18 in the supernatant were analyzed from the same number of total cells at different treatment condition. Blots are representative of three independent experiments.

It has been shown that monocytes express abundant, high affinity cell surface serpin enzyme complex (SEC) receptors which recognize a conformation specific domain of the A1AT-elastase complex [32]. To determine if conformationally-modified A1AT affects caspase-1 function we induced a conformational change in A1AT by pre-incubating human neutrophil elastase (HNE) with an equimolar concentration of A1AT to generate A1AT-HNE complexes. In this model we neutralized any excess HNE with the synthetic elastase inhibitor MeOSuccinyl-AAPV-CMK. We pretreated THP-1 cells with A1AT-HNE complexes before LPS/ATP activation. As demonstrated (**Fig. 4**) the HNE-modified form of A1AT did not suppress pro or cleaved caspase-1 nor IL-18 release. Thus, the modified form of A1AT is also incapable of preventing caspase-1 dependent inflammasomes activation in this experimental model. Furthermore, pretreating THP-1 cells with MeOSuccinyl-AAPV-CMK alone to remove monocyte derived HNE in the culture system also had no effect on THP-1 release of LPS-ATP induced IL-18 release

### Alpha-1 antitrypsin does not inhibit whole blood IL-1β release

**Fig 5:**
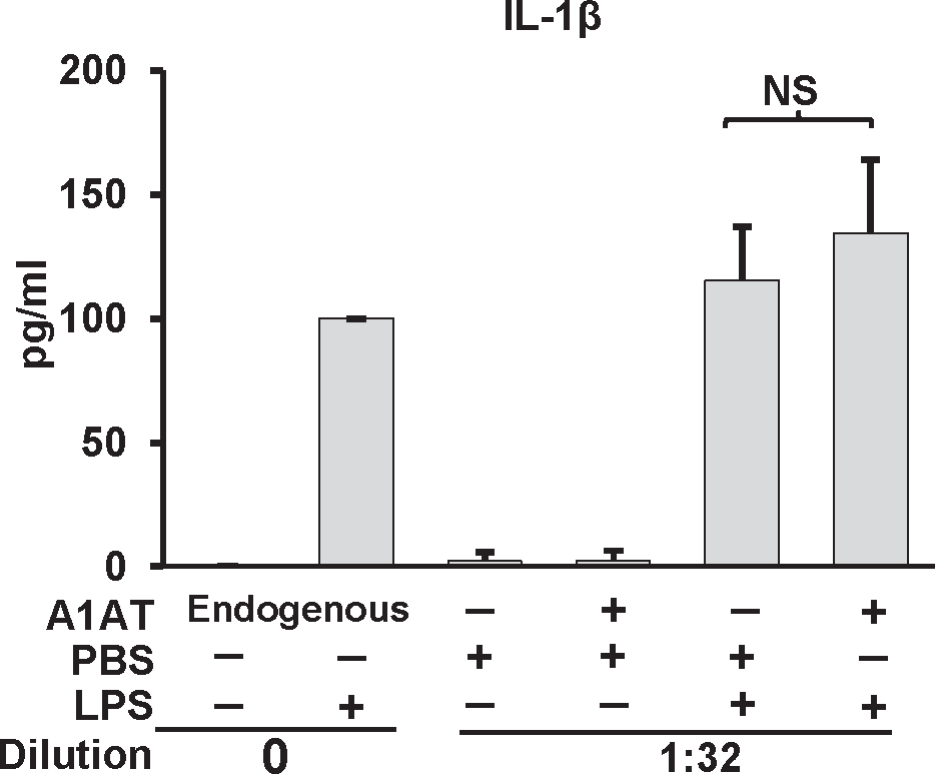
Effect of A1AT on whole blood IL-1β release . IL-1β production in whole blood cultures in response to LPS (1.0 μg/ml) was performed in the presence of endogenous A1AT (i.e., undiluted) or exogenously added A1AT (2 mg/ml) in blood diluted 1:32 with RPMI. Whole blood cultures were incubated for 18 h. After incubation, plasma supernatants were removed, and IL-1β quantified by ELISA and expressed as mean ± SD for three donors. The diluted sample result was corrected for the dilution. NS indicates no significant difference.

The release of IL-1β into whole blood samples *ex vivo* represents a useful model to study the effect of plasma derived factors such as A1AT on inflammasome function [23]. To determine if A1AT’s function requires a more physiologic model we compared normal human whole blood’s ability to release IL-1β into plasma undiluted or diluted in the presence or absence of additional exogenously applied A1AT or control human serum albumin. As shown in **Fig. 5**, physiologic concentrations of A1AT did not suppress the ability of fresh human whole blood to release IL-1β.

## Discussion

A1AT deficiency is one of the most common inherited disorders contributing to lung disease [33]. The deficiency of A1AT likely predisposes the lung to enzymatic injury from unchecked proteases such as HNE and proteinase-3 [34]. However, it has long been reported that A1AT has anti-inflammatory functions in addition to its antiprotease role. Our specific interest in the question of A1AT as a potential partner for caspase-1 was driven by our prior observation that A1AT and proIL-1β have remarkable amino acid homologies that suggest possible structural similarities [16]. Uncleaved A1AT and proIL-1β share 23% sequence identity, 23% strong amino acid similarity and 16% weak similarity for an overall shared homology of 62% [16]. We therefore sought to determine if this structural similarity might translate into functional similarity, i.e. the ability to inhibit the inflammatory enzyme, caspase-1. Indeed, several recent publications support such a potential role and are particularly intriguing [9,10,35,36]. Determining the effect of A1AT on caspase-1 is important to document since infusion therapy with purified A1AT is an established treatment modality that might be used to treat other inflammatory disorders caused by caspase-1 activation. However, using a commercial grade of A1AT we were unable to show that the serine protease inhibitor A1AT has any detectable effect on the release or activity of the cysteinyl protease, caspase-1. Neither cell free active caspase-1, live cell models of caspase-1 function, nor fresh human whole blood cell models of IL-1β release were inhibited by exogenous A1AT.

This lack of effect of A1AT on caspase-1 is not surprising. As mentioned, HNE and caspase-1 represent different classes of proteases, serine vs. cysteine. Furthermore, although both A1AT and caspase-1 can be found in the monocyte cytoplasm, caspase-1 is generated as a leaderless protein that is expressed in the cytosol [37], whereas A1AT is synthesized into the classical ER/Golgi export pathway [38]. These production differences should place these two molecules into separate cellular compartments preventing interaction.

Our conclusions however differ from recent observations that require commentary. One recent report found a difference between PiZ and PiM for IL-1β processing in monocytes after nucleofection of the respective plasmids [9]. Of interest, this work showed that THP-1 myelomonocytic cells can release PiM A1AT more readily than PiZ A1AT after nucleofection [9]. Since the PiM A1AT nucleofected cells had less IL-1β release than the PiZ cells, this was interpreted to mean that the released A1AT was responsible for the inflammasome suppression. They proposed that released A1AT may be retaken up into the cytoplasm by endocytosis thus providing direct access to caspase-1 [9]. However, it is noteworthy that blocking endocytosis did not affect A1AT’s ability to block IL-1β release [9]. Furthermore, the inability of exogenous PiM A1AT to suppress the caspase-1 function of the PiZ expressing THP-1 cells in this model agrees with our current findings. We suggest an alternative interpretation. It is conceivable in this model that the expressed PiZ protein induced a stress response due to the well-recognized protein folding problem with the PiZ molecule [39]. Misfolded A1AT might have driven cell stress-induced caspase-1 activation.

A1AT has also been previously studied using a whole blood model of endotoxin induced IL-1β release [35]. In this work, diluting whole blood into RPMI induced IL-1β release spontaneously. To determine if this dilutional effect was due to the removal of a plasma derived inhibitor (i.e. A1AT), diluted whole blood was stimulated with heat killed bacteria in the presence of infusion grade A1AT. Although A1AT was able to suppress IL-1β release, it was only effective at supra-physiological concentrations. In contrast, when we used normal concentrations of exogenous A1AT we found no effect of A1AT or human serum albumin in suppressing IL-1β release in undiluted or diluted whole blood stimulated with endotoxin. Our findings corroborate a recent study of cell-free interactions between A1AT and caspase-1 [8].

Thus, although it has been abundantly documented that A1AT has immune-modulatory functions [40–43] and there are structural similarities between A1AT and proIL-1β [16], based upon our study of distinct models that evaluated caspase-1 function at the molecular, cell-free level and at the cell level we suggest that alpha 1-antitrypsin should not be classified as a direct inhibitor of caspase-1.
